# Time-dependent Data-driven Modeling of Active Region Evolution Using Energy-optimized Photospheric Electric Fields

**DOI:** 10.1007/s11207-019-1430-x

**Published:** 2019-04-10

**Authors:** Jens Pomoell, Erkka Lumme, Emilia Kilpua

**Affiliations:** Department of Physics, University of Helsinki, P.O. Box 64, 00014 Helsinki, Finland

**Keywords:** Helicity: magnetic, Magnetic fields: corona, Corona: active, Corona: models, Magnetic fields: photosphere

## Abstract

In this work, we present results of a time-dependent data-driven numerical simulation developed to study the dynamics of coronal active region magnetic fields. The evolving boundary condition driving the model, the photospheric electric field, is inverted using a time sequence of vector magnetograms as input. We invert three distinct electric field datasets for a single active region. All three electric fields reproduce the observed evolution of the normal component of the magnetic field. Two of the datasets are constructed so as to match the energy input into the corona to that provided by a reference estimate. Using the three inversions as input to a time-dependent magnetofrictional model, we study the response of the coronal magnetic field to the driving electric fields. The simulations reveal the magnetic field evolution to be sensitive to the input electric field despite the normal component of the magnetic field evolving identically and the total energy injection being largely similar. Thus, we demonstrate that the total energy injection is not sufficient to characterize the evolution of the coronal magnetic field: coronal evolution can be very different despite similar energy injections. We find the relative helicity to be an important additional metric that allows one to distinguish the simulations. In particular, the simulation with the highest relative helicity content produces a coronal flux rope that subsequently erupts, largely in agreement with extreme-ultraviolet imaging observations of the corresponding event. Our results suggest that time-dependent data-driven simulations that employ carefully constructed driving boundary conditions offer a valuable tool for modeling and characterizing the evolution of coronal magnetic fields.

**Electronic Supplementary Material:**

The online version of this article (10.1007/s11207-019-1430-x) contains supplementary material, which is available to authorized users.

## Introduction

Coronal mass ejections (CMEs) – large-scale eruptive events originating in the solar corona (*e.g.*, Webb and Howard, 2012) – have a large impact on the plasma environment from the corona out to the heliosphere (*e.g.*, Kilpua, Koskinen, and Pulkkinen, 2017). These energetic events occur frequently, on average several times a day (week) during solar maxima (minima) (*e.g.*, Webb and Howard, 1994; Yashiro *et al.*, 2004) and are accompanied by other transient events such as flares (*e.g.*, Gosling *et al.*, 1976; Yashiro *et al.*, 2008; Murray *et al.*, 2018) and solar energetic particle events (*e.g.*, Reames, 1999). Together these phenomena are responsible for producing adverse space weather conditions throughout the heliosphere. Despite their common occurrence, the detailed physical processes that are responsible for producing CMEs are still uncertain (*e.g.*, Chen, 2011; Schmieder and Aulanier, 2012; Green *et al.*, 2018). It is, however, clear that the most energetic CMEs form in the vicinity of active regions (ARs) in the lower corona where the plasma beta (*i.e.*, the ratio of the thermal and magnetic pressure) is low. Indeed, from an energetics point of view, the amount of energy required for producing these phenomena is only available in the coronal magnetic field (*e.g.*, Low, 2001; Forbes, 2000). Consequently, characterizing the structure and dynamics of the coronal magnetic field is key to understanding the processes governing the formation and eruption of coronal mass ejections.

Progress in characterizing the coronal magnetic field has been severely hampered due to inherent difficulties in the remote observation of the magnetic field of the highly tenuous coronal plasma (*e.g.*, Cargill, 2009). As a result, efforts attempting to model the coronal magnetic field have received much attention (*e.g.*, Inoue, 2016; Wiegelmann, Petrie, and Riley, 2017). In recent years, models that incorporate data of the comparatively well-observed photospheric magnetic field have become popular. In particular, non-linear force-free field (NLFFF) extrapolations in which the coronal magnetic field is computed by solving the force-free equation have been widely adopted (*e.g.*, Wiegelmann and Sakurai, 2012). The NLFFF method can describe highly twisted fields, such as sheared arcades and magnetic flux ropes that can store free energy for powering solar eruptions.

The main shortcoming of the NLFFF extrapolation approach is the static nature of the method: each extrapolation is independent and does not account for any dynamics. Therefore, the method is not well suited for modeling the quasi-static build-up of free magnetic energy, nor is it applicable for assessing the stability of the extrapolated field, a key element when modeling CMEs and other eruptive solar events.

Only more recently have time-dependent data-driven models been constructed and employed (*e.g.*, Galsgaard *et al.*, 2015; Cheung and DeRosa, 2012; Bourdin, Bingert, and Peter, 2013; Fisher *et al.*, 2015; Jiang *et al.*, 2016; Yardley, Mackay, and Green, 2018; Hayashi *et al.*, 2018). A computationally efficient approach that has gained popularity is the time-dependent magnetofrictional (TMF) model. In the standard magnetofrictional approach (Yang, Sturrock, and Antiochos, 1986) a friction term is introduced into the magnetohydrodynamic momentum equation and in a quasi-static (and low beta) case, the velocity of the coronal plasma becomes proportional to the Lorentz force. As a result, the system relaxes towards a force-free state. However, if the photospheric boundary conditions evolve in time through which a net Poynting flux is introduced, a fully force-free state is never reached. Instead a dynamic time-dependent description of the coronal magnetic field is acquired (van Ballegooijen, Priest, and Mackay, 2000; Cheung and DeRosa, 2012). TMF has proven to be able to model the formation of coronal flux ropes (*e.g.* van Ballegooijen, Priest, and Mackay, 2000; Gibb *et al.*, 2014; Yardley, Mackay, and Green, 2018), and their lift-off and eruption (*e.g.* Mackay and van Ballegooijen, 2006; Cheung and DeRosa, 2012; Weinzierl *et al.*, 2016).

A principal problem in any time-dependent data-driven modeling, including TMF, is that of determining a realistic driving electric field. To date, most methods utilize only the line-of-sight (LOS) component of the magnetic field in deriving the electric field. Although inverting an electric field and performing data-driven modeling is possible using solely the LOS component (*e.g.* Mackay, Green, and van Ballegooijen, 2011; Cheung and DeRosa, 2012; Gibb *et al.*, 2014; Yardley, Mackay, and Green, 2018), the resulting electric field is underconstrained and thereby likely not realistic due to the significant amount of missing information. Recent studies (Schuck, 2008; Fisher *et al.*, 2010; Kazachenko, Fisher, and Welsch, 2014) imply that accurate inversion of the electric field – as well as the accurate reproduction of the related photospheric energy and helicity fluxes – requires a time series of vector magnetograms as well as photospheric plasma velocity estimates from remote sensing observations (Dopplergrams) and optical flow methods. However, the significance of the additional information provided by the vector magnetograms and photospheric plasma velocity estimates for the output of the data-driven modeling remains uncertain. By performing a parameter study using an *ad hoc* non-inductive addition to the electric field, Yardley, Mackay, and Green (2018) conclude that the additional component does not significantly alter the coronal evolution. In contrast, Cheung and DeRosa (2012), Cheung *et al.* (2015) found the non-inductive electric field to be crucial for sufficiently energizing the magnetic field for eruptive behavior to occur.

In this work, we detail a time-dependent data-driven magnetofrictional model for the evolution of active-region magnetic fields. To drive the model, we carefully construct the driving electric field by using as input a time sequence of vector magnetograms. Using this dataset as input allows us to determine the electric field only up to a scalar potential, the so-called non-inductive electric field (*e.g.*, Fisher *et al.*, 2010; Kazachenko, Fisher, and Welsch, 2014). By employing carefully constrained *ad hoc* assumptions for the latter, our inversion method reproduces the photospheric injection of magnetic energy as given by a reference estimate. This enables us to construct several electric field datasets, all consistent with the magnetogram data. By using the electric fields as input to the coronal time-dependent model, we assess the coronal response to the driving electric field. In particular, we discuss the role that energy and relative helicity play in understanding and characterizing the evolution of the coronal magnetic field.

The article is organized as follows: in Section 2, the time-dependent coronal model is detailed whereas in Section 3 we describe the method by which the observed time sequence of vector magnetograms is processed and used as boundary condition to the simulation. In Section 4, the results are presented and subsequently discussed in Section 5. In Section 6 we offer a summary and the conclusions of the work.

## Coronal Magnetic Field Modeling Method

### The Time-dependent Magnetofrictional Model

The time-dependent magnetofrictional (TMF) method models the evolution of the three-dimensional coronal magnetic field through the application of Faraday’s law,

1 $$\begin{aligned} \frac{\partial{\boldsymbol{B}}}{\partial t} = -\nabla\times{\boldsymbol{E}}. \end{aligned}$$

The electric field ${\boldsymbol{E}}$ is given by Ohm’s law, including resistivity,

2 $$\begin{aligned} {\boldsymbol{E}} = -{\boldsymbol{v}} \times{\boldsymbol{B}} + \eta \mu_{0} {\boldsymbol{J}} \end{aligned}$$

where ${\boldsymbol{v}}$ is the plasma flow velocity and the current density is given by Ampère’s law ${\boldsymbol{J}} = \nabla\times {\boldsymbol{B}}/\mu_{0}$. The resistive term is included in order to facilitate topological changes in the magnetic field in regions of high current density, and is controlled by the magnetic diffusivity $\eta$, which is chosen to be constant in this work.

In standard approaches of modeling the dynamics of plasmas such as magnetohydrodynamics (MHD), the effect of the governing forces that act on the plasma are modeled by the use of a momentum equation. In the magnetofrictional methodology, this description is considerably simplified. The essence of the method is that the velocity is set proportional to the Lorentz force. This particular choice is motivated by the fact that with this prescription the total magnetic energy in a given volume decreases monotonically in time in the absence of a net Poynting flux through the boundaries of the volume. In other words, the velocity field is such that it drives the system towards the minimum-energy force-free (but non-potential) state. This approximation can be assumed to be valid for modeling the quasi-static build-up of energy in the corona since the large wave speeds in the corona allow the magnetic field to rapidly adjust to the comparatively slow photospheric changes (*e.g.*, Wiegelmann, Petrie, and Riley, 2017). Therefore, the quiescent low-beta corona can be considered to evolve as a sequence of nearly force-free states. For eruptive events, however, employing a more complete momentum equation becomes necessary in order to accurately capture the dynamics of the eruption. Nevertheless, we posit that the overall behavior of the magnetic field (*i.e.* expulsion of excess magnetic energy *e.g.* via opening of closed fields) is adequately modeled although for instance the time-scales of the eruption are not.

In this work, the particular form for the velocity is chosen according to previous work (*e.g.* Yang, Sturrock, and Antiochos, 1986; van Ballegooijen, Priest, and Mackay, 2000; Valori, Kliem, and Fuhrmann, 2007; Jiang and Feng, 2012; Cheung and DeRosa, 2012) as

3 $$\begin{aligned} {\boldsymbol{v}} = \frac{1}{\nu} \frac{\mu_{0} {\boldsymbol{J}} \times {\boldsymbol{B}}}{B^{2}}. \end{aligned}$$

The magnetofrictional coefficient $\nu$ is assumed to be constant except in the vicinity of the photospheric boundary (the $z=0$ plane) where $1/\nu$ approaches zero smoothly:

4 $$\begin{aligned} \frac{1}{\nu} = \frac{1}{\nu_{0}} \bigl(1 - \exp(-z/L ) \bigr). \end{aligned}$$

This is done so as to ensure that the electric field at the boundary is determined entirely through the specified boundary condition and does not include a contribution from the magnetofrictional electric field (Equation 2), and is similar to that employed by Cheung and DeRosa (2012). Accordingly, only above the photosphere ($z>0$) should the magnetic field relax towards a force-free configuration.

With these choices, the magnetic field in our model is the sole dynamic variable with all other fields (${\boldsymbol{E}}, {\boldsymbol{J}}, {\boldsymbol{v}}$) computed directly from it. Note that this is in contrast to some magnetofriction-based models for computing static non-linear force-free magnetic field configurations that retain a simplified momentum equation by including the velocity as a dynamic variable (Jiang and Feng, 2012).

The speed at which the coronal magnetic field responds to a non-zero Lorentz force is determined by the magnitude of the magnetofrictional coefficient $\nu_{0}$. For the case of a static extrapolation, the time-scale of the relaxation is immaterial and restricted only by numerical stability constraints (*e.g.* Valori, Kliem, and Fuhrmann, 2007; Jiang and Feng, 2012). On the contrary, this is not the case for time-dependent simulations. Since we view Equation 3 as an approximation of a momentum equation, the range of attainable values of $\nu_{0}$ is constrained. In particular, the magnetic field is required to be able to respond to changes taking place at the imposed photospheric time scales. However, $\nu_{0}$ cannot be chosen too small (resulting in a large $|{\boldsymbol{v}}|$) since in that case the magnetic field does not have time to accumulate energy as a result of the slow photospheric changes. In this work, we set $\nu_{0} = 10^{-11}$ s m^−2^. This is the value employed by Cheung and DeRosa, 2012. The constant magnetic diffusivity is set to $\eta_{0} = 2 \times10^{8}$ m^2^ s^−1^ as in Cheung and DeRosa, 2012. This results in the ratio of the characteristic relaxation time to magnetic diffusion time to be $\eta_{0} \nu_{0} = 2 \times10^{-3}$.

### Initial and Boundary Conditions

The magnetic field at the start of the simulation (denoted by $t=0$) is chosen to be a potential field. As described in more detail in Section 4, at the chosen start time the region studied in this work contains a developed active region with significant flux that continues to evolve during the time window of the simulation. It is important to note that the potential field approximation is best suited to be used in data-driven simulations in cases where a magnetic field configuration that is initially close to potential is subsequently energized. This is only partially the case for the active region studied in this work.

The potential field is extrapolated from the normal component of the input photospheric (vector) magnetic field at the given start time. The extrapolation is done by assuming the given active-region complex to be isolated with a vanishing field at infinity. In practice, this is implemented by embedding the input magnetogram $B_{z}(x, y, z=0, t=0)$ within a much larger magnetogram consisting of $B_{z} = 0$. Then the potential field is computed assuming periodic boundary conditions in the lateral directions and vanishing horizontal components at the upper boundary which is set much higher than the chosen domain height. The final magnetic field is then selected from this spatially much larger extrapolation domain.

At the bottom of the computational domain ($z=0$) that represents the photosphere, the evolution of the simulation should be determined by the data-driven boundary conditions. Since Faraday’s law is the equation governing the evolution of the system, the boundary data should provide the information required to compute the right hand side of Equation 1. As discussed by Cheung and DeRosa (2012), if an Ohm’s law of the form of Equation 2 is assumed to be valid also in the photosphere, then it is sufficient to give only the horizontal components of the electric field ${\boldsymbol{E}} _{\mathrm{h}}$ as a boundary condition. This arises from the fact that the fields ${\boldsymbol{v}}$, ${\boldsymbol{B}}$ and ${\boldsymbol{J}}_{\mathrm{h}}$ required for determining ${\boldsymbol{E}}_{\mathrm{h}}$ also completely determine the normal component of the electric field. Moreover, specifying the horizontal components of the electric field ${\boldsymbol{E}}_{\mathrm{h}}$ at $z=0$ determines the evolution of the normal component of the magnetic field $B_{z}(x,y,z=0,t)$ completely. This is advantageous, as $B_{z}$ is in practice the quantity determined to the highest degree of accuracy from the remote sensing observations. The horizontal components ${\boldsymbol{E}} _{\mathrm{h}}$ are provided by an inversion procedure using a time-sequence of vector magnetograms as input. This process is detailed in Section 3.

At the lateral and top sides the simulation domain is considered to be open, *i.e.*, plasma is allowed to freely pass through these boundaries. In practice, this is implemented by extrapolating the magnetic, velocity and current density fields from the simulation cells located closest to the domain boundary out to the edges of the domain.

### Numerical Implementation

The TMF system is solved using a finite difference approach employing a staggered grid. The primary variable of the simulation, the magnetic field, is located in a staggered fashion at the centers of the faces of each cell (the so-called Yee-grid, Yee, 1966). Thus, for a cell with the center at coordinate $(x_{i}, y_{j}, z_{k})$, the $x$-component of the magnetic field is located at the cell faces with normals in the $x$-direction, *i.e.* at coordinates $(x_{i} \pm\Delta x/2, y_{j}, z_{k})$ with $\Delta x$ the length of the cell in the $x$-direction. These components are denoted $B_{x;i\pm1/2,j,k}$. Similarly, the $y$-component of the magnetic field is located at $(x_{i}, y_{j} + \Delta y/2, z_{k})$ and is denoted by $B_{y; i, j \pm1/2, k}$. The electric field and current density are co-spatial at the centers of the cell edges. For instance, the $x$-component of the electric field is located at $(x_{i} \pm\Delta x/2, y_{j} \pm\Delta y/2, z_{k})$. This grid arrangement is commonly used in numerical MHD algorithms that employ the constrained transport method (see Kissmann and Pomoell, 2012 and the references therein).

The current density is computed directly from the magnetic field employing second-order finite differences, *e.g.* the $z$-component of the current density is computed as

5 $$\begin{aligned} \mu_{0} J_{z; i-1/2, j-1/2, k} = \frac{B_{y; i, j-1/2, k} - B_{y; i-1, j-1/2, k}}{\Delta x} - \frac{B_{x;i-1/2,j,k} - B_{x;i-1/2,j-1,k}}{ \Delta y} \end{aligned}$$

where the index $(i, j, k)$ again refers to the cell center position, and a half index refers to the position a half-cell distance away from the cell center.

While the resistive contribution to the electric field can immediately be obtained from the edge-staggered current density, computation of the Lorentz force and thereby the TMF velocity necessarily requires interpolating the quantities to a common location. We follow an approach similar to that presented in van Ballegooijen, Priest, and Mackay (2000), *i.e.* the magnetic field and current density are interpolated via simple averaging of the nearest data points to cell corner positions. The magnetofrictional velocity, Equation 3, is then computed directly from the interpolated components. A complication that arises in the practical computation of the velocity is that in localized regions of small magnetic field (*e.g.* in the vicinity of null points, current sheets etc.) the velocity can become excessively large. To remedy this, rather than specifying an arbitrary floor value for the magnetic field, a filter is applied to the velocity so that the final velocity vector is computed as

6 $$\begin{aligned} {\boldsymbol{v}} = f\bigl(|{\boldsymbol{v}}_{\mathrm{MF}}|\bigr) {\boldsymbol{v}}_{ \mathrm{MF}} \end{aligned}$$

where ${\boldsymbol{v}}_{\mathrm{MF}}$ refers to the velocity obtained using Equation (3) and $f(v) = \tanh(\xi)/ \xi$ is the chosen filtering function where $\xi= \epsilon+ v/v _{\mathrm{lim}}$ where $\epsilon= 10^{-12}$ is a small number to avoid division by zero. This choice results in ${\boldsymbol{v}} \approx {\boldsymbol{v}}_{\mathrm{MF}}$ for $v_{\mathrm{MF}} < v_{\mathrm{lim}}$ while ${\boldsymbol{v}} \approx v_{\mathrm{lim}} \hat{{\boldsymbol{v}}} _{\mathrm{MF}}$ for $v_{\mathrm{MF}} > v_{\mathrm{lim}}$. Thus, the filtering returns the magnetofrictional velocity for speeds smaller than the limiting speed, whereas for larger speeds the direction of the velocity is retained but the magnitude is scaled to that of the given limiting speed. In this work we set the limiting speed $v_{ \mathrm{lim}} = 30$ km s^−1^. Velocities larger than that can be deemed unreasonable in the magnetofrictional description.

Once the velocity and magnetic fields have been computed at the corners of each cell, the edge-centered electric field needs to be computed. For this procedure we employ the method presented by Evans and Hawley (1988) (see their Section 4.d). The method employs upwind biasing based on the flow velocity (here the magnetofrictional velocity) together with slope limiting when computing the convection electric field.

With the electric determined at the edge-centers, the time-evolution of the magnetic field is then given by Faraday’s law discretized as

7 $$\begin{aligned} \partial_{t} B_{z; i-1/2, j-1/2, k} = \frac{E_{y; i, j-1/2, k}-E_{y; i-1, j-1/2, k}}{\Delta x} - \frac{E_{x;i-1/2,j,k} - E_{x;i-1/2,j-1,k}}{ \Delta y}. \end{aligned}$$

The semi-discrete system above can be evolved forward in time with in principle any integrator of choice. Commonly, in the MHD case, a second or third order Runge–Kutta-based scheme is employed. However, in test runs we found little benefit of using a higher-order method. Thus, in this work, all the results presented employ a first-order forward Euler time-stepping scheme.

It is important to note that although the system of equations appear to be hyperbolic (as is the case for MHD), the choice of the magnetofrictional velocity in fact makes the equations parabolic (Craig and Sneyd, 1986). As a consequence, the explicit time-stepping scheme requires that the maximum stable time step scales with the mesh spacing squared. In accordance with this, we use $\Delta t = C \Delta ^{2} \nu_{0}$ with $C$ a Courant-like constant of the order of unity and $\Delta$ the grid spacing to specify the size of the time step $\Delta t$.

The staggered co-location of the variables on the grid is advantageous as it preserves to machine accuracy properties of the vector fields at the discrete level. For instance, Faraday’s law preserves exactly the divergence of the magnetic field of the initial state from which the computation is started. For this reason, it is important to compute the initial potential magnetic field such that it is both divergence-free and current-free up to machine precision. In practice, this entails solving a discretized Laplace equation exactly. To achieve this, we utilize the elliptic solver routines available in the FISHPACK library (Swarztrauber and Sweet, 1975). The staggered co-location of the variables also facilitate the implementation of the boundary conditions as the horizontal components of the electric field are the only ones that need to be specified in the numerical solution scheme. To summarize, the two horizontal electric field components as a function of time as well as the normal component of the initial magnetic field are required to be given as input at a common plane. This plane constitutes the data-driven input data plane, and is co-spatial with the $z$-directed face of the lowermost cells in the simulation (*i.e.* at coordinate $z_{k=-1/2}$ with $k=0$ indicating the index of the first cells in the $z$-direction).

## Data-driven Photospheric Boundary Conditions

In addition to a dynamic model of the coronal magnetic field, conducting time-dependent data-driven modeling requires a second main ingredient, namely evolving boundary conditions determined from available observations. As discussed in the previous section, the model considered in this work requires the horizontal components of the electric field at the lower boundary as a function of time, *i.e.*

8 $$\begin{aligned} {\boldsymbol{E}}_{\mathrm{h}}(x, y, z=0, t) = E_{x}(x, y, z=0, t) \hat{{\boldsymbol{x}}} + E_{y}(x, y, z=0, t) \hat{{\boldsymbol{y}}} \end{aligned}$$

to be completely given as input data. The method by which these are determined is detailed in this section. For brevity in the following we do not include the notation that explicitly states that the fields are evaluated at the height $z=0$. Thus, it is important to note that the fields considered in this section are spatially two-dimensional.

### Electric Field Inversion Method

To determine the data-driven electric field, we employ the method presented in our previous work (Lumme, Pomoell, and Kilpua, 2017). The approach follows the work of Fisher *et al.* (2010), Fisher, Welsch, and Abbett (2012), Kazachenko, Fisher, and Welsch (2014), Kazachenko *et al.* (2015) in that the total electric field is constructed as a sum of an inductive electric field ${\boldsymbol{E}}_{\mathrm{I}}$ and a non-inductive component given by a scalar potential $\psi$:

9 $$\begin{aligned} {\boldsymbol{E}} = {\boldsymbol{E}}_{\mathrm{I}} - \nabla\psi. \end{aligned}$$

Given the time-evolution of the photospheric magnetic field, the inductive electric field is completely determined by $\partial_{t} {\boldsymbol{B}} = -\nabla\times{\boldsymbol{E}}_{\mathrm{I}}$. In practice, this equation is uncurled by using a poloidal–toroidal decomposition of the magnetic field which results in Poisson problems to be solved. One advantage of this approach when employed together with a staggered discretization is that a solution procedure for computing the inductive electric field can readily be constructed so that the given evolution of the normal component of the magnetic field $B_{z}$ is reproduced exactly (for details, see Lumme, Pomoell, and Kilpua, 2017). This is in contrast to other methods (*e.g.* Schuck, 2008; Hayashi *et al.*, 2018) in which the observed $B_{z}$ is only approximately reproduced.

While the inductive electric field can be directly computed from a set of photospheric magnetic field measurements, determining the non-inductive electric field requires additional observational data, such as plasma flow estimates from Doppler measurements and optical flow tracking (Kazachenko, Fisher, and Welsch, 2014). On the other hand, a simpler approach, which does not require additional data, can be adopted, in which the non-inductive electric field is assumed to fulfill a given functional form. A rigorous way of doing this was presented in Lumme, Pomoell, and Kilpua (2017), and it is the approach we adopt in this work.

The method to determine the non-inductive electric field employed in this study requires two ingredients. First, a functional form for the non-inductive component. As in Lumme, Pomoell, and Kilpua (2017), we consider three distinct assumptions:

10 $$\begin{aligned} \nabla^{2} \psi =& 0, \end{aligned}$$

11 $$\begin{aligned} -\nabla_{\mathrm{h}}^{2} \psi =& \nabla_{\mathrm{h}} \cdot{\boldsymbol{E}}_{\mathrm{h}} = \Omega B_{z}, \end{aligned}$$

12 $$\begin{aligned} -\nabla_{\mathrm{h}}^{2} \psi =& \nabla_{\mathrm{h}} \cdot{\boldsymbol{E}}_{\mathrm{h}} = U ( \nabla\times{\boldsymbol{B}})_{z}, \end{aligned}$$

where $\Omega$ and $U$ are constants (both temporally and spatially) to be specified. We refer to these three assumptions as the zero-, $\Omega$- and $U$-assumption, respectively. The latter two forms were suggested by Cheung and DeRosa (2012) and Cheung *et al.* (2015), respectively, in order to ensure sufficient injection of free magnetic energy to their TMF simulation domain, and to produce a coronal evolution in better agreement with the observed dynamics. In highly idealized settings these assumptions can be shown to correspond to the ideal electric field response of a rigidly rotating axisymmetric vertical flux tube ($\Omega$-assumption) and to the uniform vertical emergence of an axisymmetric flux tube with uniform twist ($U$-assumption) (see also Lumme, Pomoell, and Kilpua, 2017).

The second ingredient that is required is an independent reference metric that allows one to determine the values for the free parameters $\Omega$ and $U$. For this purpose, we require that the magnetic energy injection as computed from the total electric field (Equation 9) should be consistent with the estimate computed from the DAVE4VM optical flow-based electric field estimate (Schuck, 2008; Liu and Schuck, 2012):

13 $$\begin{aligned} {\boldsymbol{E}}_{\mathrm{DAVE4VM}} = -{\boldsymbol{V}}_{\mathrm{DAVE4VM}} \times{\boldsymbol{B}}, \end{aligned}$$

where ${\boldsymbol{V}}_{\mathrm{DAVE4VM}}$ is the DAVE4VM velocity estimate, based on the vector magnetogram input (the unmasked version of the ${\boldsymbol{B}}^{\mathrm{opt}}$ dataset defined in Section 3.2) to the DAVE4VM code (see Lumme, Pomoell, and Kilpua, 2017, for details of how we use DAVE4VM). This kind of DAVE4VM velocity/electric field estimate has been shown to yield reasonable energy flux estimates when compared to other methods (Kazachenko, Fisher, and Welsch, 2014; Kazachenko *et al.*, 2015; Lumme, Pomoell, and Kilpua, 2017). However, the DAVE4VM electric field itself cannot be used in data-driven modeling due to fact that the electric field in Equation 13 fulfills Faraday’s law only in the least squares sense, resulting in a loss of inductivity of the electric field when applied to real magnetogram observations (see Schuck, 2008; Lumme, Pomoell, and Kilpua, 2017, for details).

The energy injection is computed by integrating the Poynting flux over the magnetogram area and over time:

14 $$\begin{aligned} E_{\mathrm{M}}(t) = \int_{0}^{t} \mathrm{d}t' \int\mathrm{d}A \, \frac{1}{\mu_{0}} ({\boldsymbol{E}} \times{\boldsymbol{B}}) \cdot\hat{{\boldsymbol{z}}} . \end{aligned}$$

Thus, determining the non-inductive part is an optimization problem in which the free parameters $\Omega$ and $U$ are to be found such that the given DAVE4VM-based energy injection be reproduced as closely as possible. The zero-assumption, the simplest possible assumption for the non-inductive part, is retained in order to assess the degree to which the photospheric fluxes can be obtained by neglecting the non-inductive component. It is important to note that the energy injection is estimated globally, *i.e.*, using a single value for all magnetograms. This is in accordance with the simplification that the parameters $\Omega$ and $U$ are assumed to be constants both in time and space.

Finally, note that in computing the electric field using DAVE4VM no mask is employed (the input consists of the unmasked version of ${\boldsymbol{B}} ^{\mathrm{opt}}$; see Section 3.2). However, when computing the energy and relative helicity injections (Equations 14 and 18), the magnetic field is masked (see step 4 in the first processing stage presented in Section 3.2). Thus, the magnetic field and vector potential components used in computing the vertical Poynting and relative helicity fluxes are identical, and only the electric field for the different inversions is different.

### Vector Magnetogram Processing Procedure

The electric field inversion procedure outlined above requires as input a sequence of photospheric vector magnetogram maps from which the required quantities be computed. In this work we utilize vector magnetogram datasets derived from observations by the *Helioseismic and Magnetic Imager* (HMI) (Schou *et al.*, 2012; Scherrer *et al.*, 2012) on the *Solar Dynamics Observatory* (SDO) (Pesnell, Thompson, and Chamberlin, 2012) described in detail by Hoeksema *et al.* (2014). Rather than using available HMI datasets directly, we process the vector magnetogram data using a pipeline described below in order to make the datasets more suitable as input to the TMF simulation.

The processing of the vector magnetogram data proceeds in two distinct stages. In the first, the data are processed according to the steps described in detail in Lumme, Pomoell, and Kilpua (2017). To summarize, the main parts of the pipeline are:

i) Full-disk disambiguated vector magnetograms (JSOC hmi.B_720s data product, (Hoeksema *et al.*, 2014)) for the selected time period are downloaded.
ii) A region that encloses the region of interest is selected and this region is tracked in the full-disk magnetograms over its disk transit. Bad pixels (Hoeksema *et al.*, 2014) are filtered from the cutouts and the data are reprojected to a local Cartesian system using Mercator map projection centered at the region of interest.
iii) Spurious temporal flips in the magnetic field azimuth are removed from this reprojected and remapped magnetogram time series using a regularization procedure following Welsch, Fisher, and Sun (2013).
iv) Weak field regions that are largely dominated by noise are masked to zero using a threshold on the absolute value of the magnetic field of 250 Mx cm^−2^ similar to Kazachenko *et al.* (2015).

The sequence of vector magnetic field maps obtained at this stage (denoted by ${\boldsymbol{B}}^{\mathrm{opt}}$) are then used in the electric field inversion procedure to find the optimal values for the free parameters $\Omega$ and $U$. The energy-optimized electric field datasets thus obtained are denoted ${\boldsymbol{E}}^{\mathrm{opt}}$. These are collectively denoted as the $\mathrm{opt}$-dataset.

In a second stage of processing, the ${\boldsymbol{B}}^{\mathrm{opt}}$ dataset is further prepared so as to produce a dataset suitable for the data-driven simulation, ${\boldsymbol{B}}^{\mathrm{sim}}$ and ${\boldsymbol{E}}^{\mathrm{sim}}$. We denote this the $\mathrm{sim}$-dataset. The pipeline in this final stage is as follows:

i) The magnetic field dataset ${\boldsymbol{B}}^{\mathrm{opt}}$ is smoothed in space and time using a Gaussian filter with a standard deviation of 4 pixels (4×364 km) in spatial domain and 4 time steps (4×12 minutes) in time domain.
ii) The magnetic field data is rebinned to a resolution four times coarser (*i.e.* $\approx2''$ or 1456 km) than the input dataset.
iii) At the outermost 20 pixels of the magnetogram, the magnetic field values are smoothly scaled (using a $\tanh()$ tapering profile) so that the values are exactly zero at the edge of the magnetogram.
iv) The flux given by the normal component $B_{z}$ is balanced to zero using a method in which each positive-valued $B_{z}$-pixel is multiplied by a constant $B^{+}$ and each negative value by a constant $B^{-}$ with the constants chosen to balance the net flux but leave the unsigned flux unchanged.

The principal processing step in this second stage is that of rebinning the magnetogram data to a coarser resolution in order to reduce the computational resources required by the simulation. However, in the process we also choose to smooth the data. The smoothing helps in mitigating spurious large values in the derivatives of the magnetic field due to errors in measurements as well as masking of the weak field. In addition, the smoothing makes the solution less dependent on the stabilizing non-linear diffusive properties of the numerical scheme. Similar smoothing is often employed in data-driven coronal modeling, both in time-dependent simulations (*e.g.*, Jiang *et al.*, 2016) as well as static extrapolations (*e.g.*, Wiegelmann, Inhester, and Sakurai, 2006). The tapering of the magnetic field is done in order to avoid dynamics at the edges of the simulation domain that cannot be resolved due to the proximity of the edge of the domain. Note that this step, as well as the balancing of the flux, is consistent with the basic assumption that the studied region is isolated from the surroundings. After these processing steps, the electric field ${\boldsymbol{E}}^{\mathrm{sim}}$ finally used as input to the simulation is computed using the obtained ${\boldsymbol{B}}^{\mathrm{sim}}$ dataset and the values for $\Omega$ and $U$ determined previously using the $\mathrm{opt}$-dataset. As a result, the energy injection in the $\mathrm{sim}$-dataset is altered from that of the optimized $\mathrm{opt}$-dataset.

## Results: Data-driven Modeling of NOAA AR 11504

In this section, we present the results from our time-dependent data-driven modeling approach applied to NOAA active region (AR) 11504. On June 14 2012 the active region experienced an eruption producing a M1.9 class flare (peak flux at 12:52 UT) and a full halo coronal mass ejection that was first detected at 13:48 UT (onset time as provided by CACTUS, Robbrecht and Berghmans, 2004). Approximately 2.5 days later *in situ* spacecraft data in the vicinity of Earth show an ejecta with clear hallmarks of a flux rope magnetic field structure. Observational details of the event, relating to the formation of the flux rope, its properties as well as interplanetary propagation have been studied by Palmerio *et al.* (2017), James *et al.* (2017), Srivastava, Mishra, and Chakrabarty (2018).

To model the event, we select the time interval from June 11 00:00 UT to 12:00 UT on June 15, 2012. This interval captures the main emergence of new flux into the active region (James *et al.*, 2017). While the AR is also active prior to June 11 (*e.g.* M-class flares on June 9 and 10), the degradation of the quality of the vector magnetogram data towards the limb (Hoeksema *et al.*, 2014; Sun and Norton, 2017) does not allow to include this phase in the modeling.

**Figure 1:**
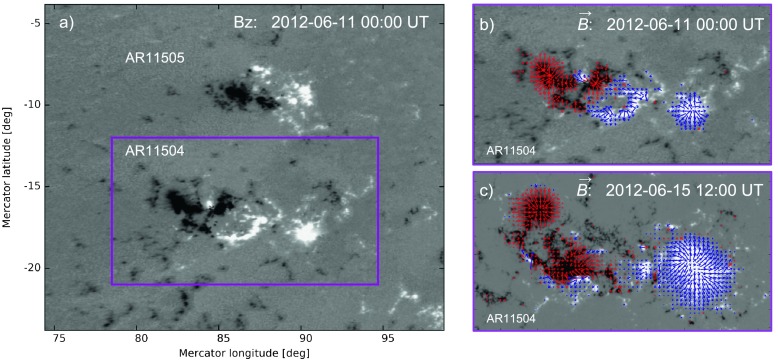
Overview of the full extent of the magnetogram used in the analysis and simulation that includes ARs 11504 and 11505. The *magenta box* indicates the region approximately designated as AR 11504. (**a**) shows the normal component of the full resolution unmasked HMI magnetic field for the time corresponding to the initial state of the simulation. (**b**) and (**c**) show the region indicated by the *magenta box* in panel **a**), with panel **b**/**c** depicting the photosphere on 2012-06-11 00:00/2012-06-15 12:00 UT, respectively. The *arrows* depict the horizontal component of the field and are colored by the polarity (*red* = negative) of the field. The grayscale of the normal component is saturated at $\pm0.1~\mbox{T} = 1000$ G.

Figure 1 (panel a) shows the full reprojected magnetogram cutout patch used in our analysis and simulation on June 11 00:00 UT (see Section 3.2). The magenta box indicates the region that corresponds approximately to the region designated as NOAA AR 11504. Panels b and c show the vector magnetic field for the boxed region at the beginning (panel b) and end (panel c) of the selected time window. Note that the full cutout (panel a) used in the analysis as well as in the simulation contains also the smaller active region (NOAA AR 11505) to the north. For this magnetogram, the ratio of the signed to the unsigned magnetic flux is $\approx-17 \%$ at the start of the simulation time window and decreases in a linear fashion reaching $\approx-2 \%$ on June 15 00 UT.

### Photospheric Energy and Relative Helicity Injection

Using the selected cutout and time window, the procedure of determining the electric field that approximates the energy injection as provided by DAVE4VM (see Section 3.1) was carried out. The resulting free parameters of the non-inductive electric field thus determined were found to be

15 $$\begin{aligned} \Omega =& (0.141 \pm0.01) \times2\pi~\mbox{day}^{-1}, \end{aligned}$$

16 $$\begin{aligned} U =& (160\pm5)~\mbox{m}\,\mbox{s}^{-1} \end{aligned}$$

where the accuracy reflects the size of the increments used when finding the optimal values.

**Figure 2:**
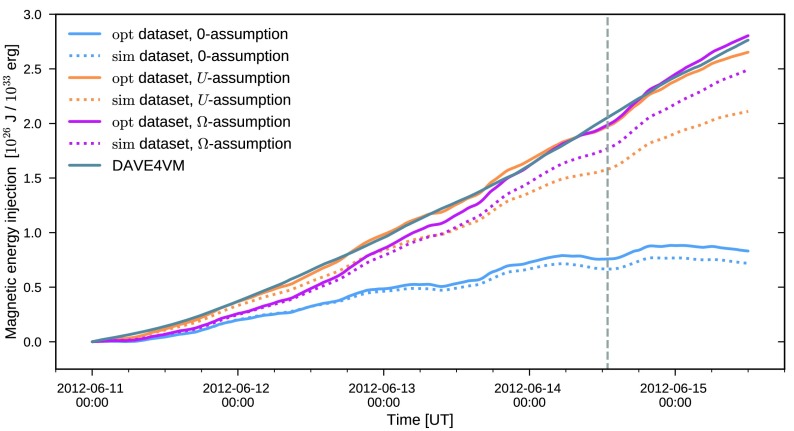
Photospheric magnetic energy injection computed using Equation 14. The *solid curves* show the energy injection computed using the dataset obtained through optimization ($\mathrm{opt}$-dataset), while the *dotted curves* use the dataset further processed and used as input to the coronal simulation ($\mathrm{sim}$-dataset). The *blue/magenta/yellow curves* correspond to electric field inversion using the zero/$\Omega$/$U$-assumptions (Equations 10/11/12 for the non-inductive component), respectively. The *vertical dashed line* indicates the time of the M-class flare that occurred on June 14.

In Figure 2 the injection of magnetic energy as a function of time, computed using Equation (14), for the energy-optimized electric fields is shown as solid curves. The two electric field inversions that have a non-zero non-inductive component succeed in capturing the target energy injection computed using DAVE4VM (gray solid curve). In particular, the electric field employing the $U$-assumption (yellow solid curve) follows the DAVE4VM time evolution very closely, while the $\Omega$-assumption (magenta solid curve) initially slightly underestimates the energy injection until approximately 18:00 on June 13, while slightly overestimating the injection during June 15. In contrast, the assumption that sets the non-inductive component to zero (blue solid curve) consistently underestimates the target energy injection. As a result, the total magnetic energy input during the considered four and a half days is significantly, by a factor $\approx3.3$, lower when neglecting the non-inductive component (being $8.3 \times10^{25}$ J with zero non-inductive component and $2.8 \times10^{26}$ J for the DAVE4VM estimate).

Figure 2 also plots as dotted lines the energy injection for the $\mathrm{sim}$-dataset $({\boldsymbol{B}}^{ \mathrm{sim}}, {\boldsymbol{E}}^{\mathrm{sim}})$ that was further processed for use as input to the coronal simulation (as described in Section 3.2). Although the trend of the energy injection using the two datasets closely follow each other, the $\mathrm{sim}$-dataset consistently exhibits a lower energy injection. The level of underestimation is also different for the three assumptions for the non-inductive electric field. When neglecting the non-inductive component, the total energy injected is reduced by $13 \%$ when using the $\mathrm{sim}$-dataset, while for the case of the $U$-assumption the corresponding number is $20 \%$.

**Figure 3:**
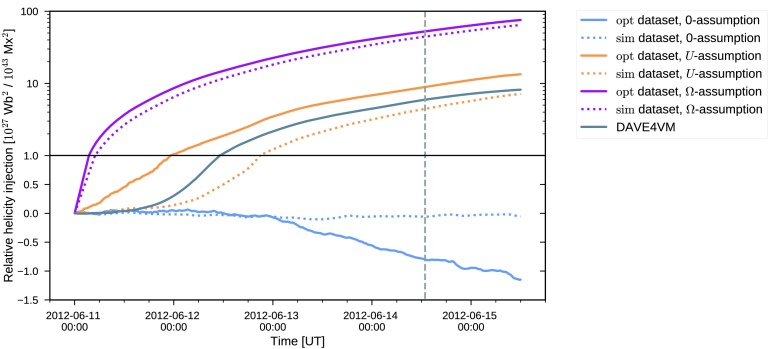
Photospheric relative helicity injection computed using Equation 18. The *curve legend* is identical to Figure 2. Note that the *lower part of the plot* employs a linear scale, while the *upper* employs a logarithmic one. The *solid horizontal line* indicates the separation of the two scales.

In addition to the injection of magnetic energy, the datasets also allow to compute the injection of relative helicity, *i.e.*, the helicity in excess of the helicity of the potential field. For a given volume, the relative helicity is defined as (Berger and Field, 1984; Finn and Antonsen, 1985)

17 $$\begin{aligned} H_{\mathrm{R}} = \int\mathrm{d}V \,({\boldsymbol{A}}+{\boldsymbol{A}}_{\mathrm{p}}) \cdot({\boldsymbol{B}}-{\boldsymbol{B}}_{\mathrm{p}}) \end{aligned}$$

where ${\boldsymbol{B}} = \nabla\times{\boldsymbol{A}}$ and the subscript $\mathrm{p}$ refers to the corresponding potential field. The injection of relative helicity from the photosphere can be computed by integrating the flux of relative helicity in time (Berger and Field, 1984),

18 $$\begin{aligned} H_{\mathrm{R}}(t) = \int_{0}^{t} \mathrm{d}t' \int\mathrm{d}A \,\bigl[-2({\boldsymbol{A}} _{\mathrm{p}} \times{ \boldsymbol{E}}) \cdot\hat{{\boldsymbol{z}}} \bigr] \end{aligned}$$

where the expression for the flux of the relative helicity follows from utilizing a specific choice for the gauge of the magnetic vector potential ${\boldsymbol{A}}_{\mathrm{p}}$ (see, *e.g.*, Pariat *et al.*, 2015). In Figure 3, the resulting relative helicity injection is shown.

In contrast to the magnetic energy injection, the relative helicity injection for the three electric field inversions are vastly different. For the electric field inverted using the $\Omega$-assumption, the total relative helicity injected during the five days considered is ≈5.6 times larger than for the $U$-assumption. The $\Omega$-assumption case also drastically overestimates the DAVE4VM result, by a factor of ≈9.2 while the $U$-assumption case results in a smaller overestimation of ≈64%. The large overestimation of the relative helicity injection for the $\Omega$-assumption arises from the fact that the non-inductive component injects positive (since $\Omega> 0$) relative helicity for all pixels of each magnetogram, as shown by Lumme, Pomoell, and Kilpua (2017). When the non-inductive component is neglected, the injection is significantly smaller compared to the other inversion results. From the beginning of June 13, the trend is even opposite to the others as the injection becomes negative and the relative helicity thus diminishes. Computing the relative helicity injection using the $\mathrm{sim}$-dataset shows a similar change as in the case of the magnetic energy: the injection is consistently reduced compared to the $\mathrm{opt}$-dataset. Also, the changes are again not uniform across the three assumptions. For the $U$-assumption the relative helicity injection is reduced by $47 \%$ whereas the $\Omega$-assumption experiences a drop of $15 \%$. For the zero-assumption, the relative helicity injection remains small throughout the time period.

### Magnetic Energy and Relative Helicity in the Corona

We ran the time-dependent data-driven coronal simulation using the three electric field datasets computed from the $({\boldsymbol{B}}^{ \mathrm{sim}}, {\boldsymbol{E}}^{\mathrm{sim}})$ data (see Sections 3.1 and 3.2 for details). The three simulations are denoted the zero, $\Omega$ and $U$-simulations corresponding to the inversion using the assumption with the same name.

**Figure 4:**
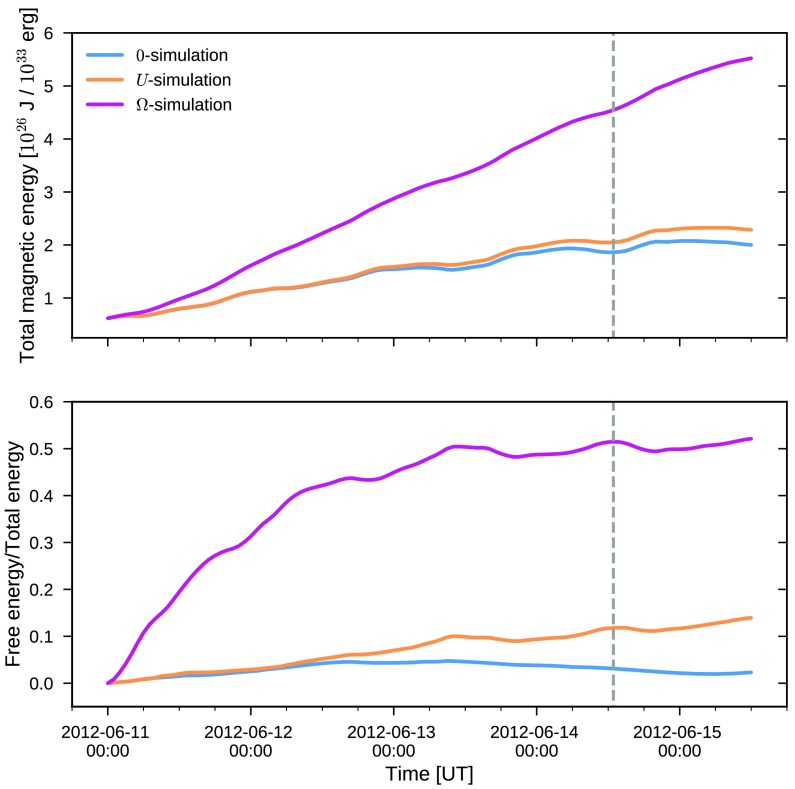
*Upper*: Magnetic energy in the coronal simulation volume as a function of time. *Lower*: Ratio of free magnetic energy to the total magnetic energy in the simulation as a function of time.

While the two-dimensional photospheric maps allow to compute the injection of energy, the simulation allows one to compute the time-dependent energization of the three-dimensional coronal magnetic field. In the upper panel of Figure 4, we plot as a function of time the total magnetic energy in the simulation volume computed as

19 $$\begin{aligned} E = \int\mathrm{d}V \,\frac{B^{2}}{2 \mu_{0}}. \end{aligned}$$

The figure shows that the total coronal energy for the zero-simulation (*i.e.*, neglecting the non-inductive electric field) follows that of the $U$-simulation: rising from $6.2 \times10^{25}$ J contained in the potential field at the start of the simulation to $2.0 \times10^{26}$ J for the former and $2.3 \times10^{26}$ J for the latter. However, in the $\Omega$-simulation, the magnetic energy attains considerably larger values reaching $5.5 \times10^{26}$ J at the end of the run. Also the rate at which the energy increases is significantly different. As a result, already only after about one and half day from the start of the simulation (at 14:00 on June 12), the $\Omega$-simulation contains more energy than the $U$-simulation attains during the entire simulation time. It is notable that the behavior of the energy content for all three simulations is in contrast to the injection of energy (Figure 2) which by construction was similar for the inversions including a non-inductive component and smaller when neglecting it.

In the lower panel of Figure 4, the ratio of the free magnetic energy (*i.e.*, the energy in excess of the potential energy),

20 $$\begin{aligned} E_{\mathrm{free}} = \int\mathrm{d}V\, \frac{B^{2} - B_{\mathrm {p}}^{2}}{2 \mu _{0}}, \end{aligned}$$

to the total energy is plotted. Initially for all three simulations the free magnetic energy increases monotonically, however, with the increase being significantly faster for the $\Omega$-simulation. At later times, the simulations exhibit a different behavior. For the $\Omega$-simulation the free energy increase saturates around mid-June 13 after the rapid initial rise. While the free energy in the $U$-simulation continues to slowly increase throughout the whole run, the opposite takes place in the zero-simulation at around June 13 after which the free energy ratio starts to decrease. At the end of the run, only $2.3 \%$ of the energy in the zero-simulation is contained as free energy, whereas in the $U$-simulation the corresponding number is $14 \%$. In the case of the $\Omega$-simulation, the ratio of free energy saturates after the quick increase at roughly $50 \%$. Note, however, that the amount of free energy continues to increase since the total energy increases monotonically throughout the simulated interval (upper panel of Figure 4).

**Figure 5:**
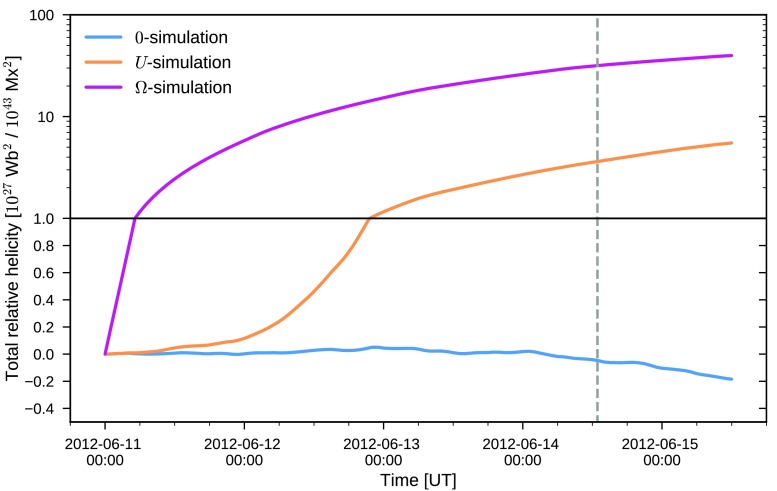
Total relative helicity in the coronal volume plotted similarly to Figure 3.

In addition to the total energy contained in the simulation, we also compute the total relative helicity content in the simulation volume (Equation 17) employing the method described in Valori, Démoulin, and Pariat (2012). The results of this computation are shown in Figure 5. For the $U$-simulation, the relative helicity shows a slow increase until June 12 after which the rate of relative helicity accumulation significantly increases. In contrast, the $\Omega$-simulation shows a much faster increase already at the beginning of the simulation. At 00:00 June 12, the $\Omega$-simulation contains the equal amount of relative helicity as the $U$-simulation at the end of the run. In contrast, the relative helicity in the zero-simulation remains small, and becomes negative after early on June 14.

### Coronal Magnetic Field Evolution

We study the evolution of the coronal magnetic field using the three electric field datasets. To visualize the morphology of the coronal magnetic field we trace a set of field lines starting from the same points at each time and for the three simulations, with each field line given a different color. The set of seed points from which the tracing is started is chosen to be a plane located 5 Mm above the photosphere covering both active regions.

**Figure 6:**
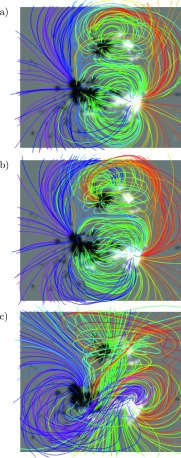
Structure of the magnetic field at 00:00 UT on June 13. (**a**) shows the zero-simulation, (**b**) the $U$-simulation and (**c**) the $\Omega$-simulation. The grayscale plane depicts $B_{z}(x,y,z=0)$ and is saturated at $\pm500$ G.

The resulting field lines are shown in Figure 6 at midnight on June 13, two days after the start of the simulation. Panels a and b depict the field lines for the zero and $U$-simulations, respectively. Overall, the large-scale structure of the magnetic field is similar for the two simulations, especially for the larger loops. For the magnetic field lines straddling the polarity inversion lines, a larger difference is observed, with the field lines appearing more sheared in the $U$-simulation. For the $\Omega$-simulation (panel c), however, the magnetic field structure differs substantially from the two other simulations. The field lines traced by the dark blue and magenta curves indicate the existence of a large coherent flux rope structure in the southern active region (AR 11504) connecting the northern edges of the main polarities. Also the smaller northern active region appears very different from the two other simulation runs. A movie of Figure 6 is available in the online supplementary material.

#### Flux Rope Properties and Eruption

**Figure 7:**
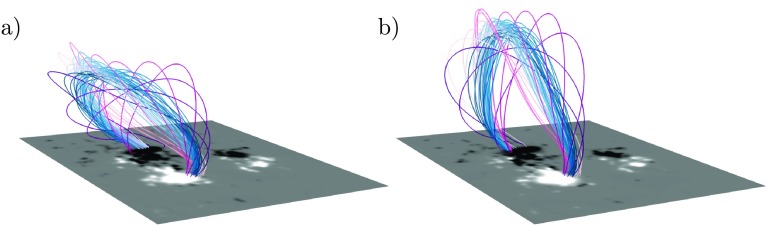
Magnetic field lines of the flux rope as it rises in the $\Omega$-simulation. (**a**) The flux rope at 00:00 June 13. (**b**) Flux rope at 10:00 June 13.

As noted in the previous subsection, the $\Omega$-simulation produces a twisted flux rope magnetic field in AR 11504. While the structure is clearly present on June 13 (Figure 6c), highly twisted S-shaped field lines straddling the polarity inversion line are already visible at June 12, 12:00 UT followed shortly by the first indications of a coherent flux rope. Approximately this time, the flux rope starts to steadily rise. This is depicted in Figure 7, which shows selected field lines of the flux rope at two different times, 00:00 UT (left panel) on June 13 (same time as in Figure 6) and ten hours later. As in Figure 6, the color of the field lines are chosen in order to more clearly visualize the magnetic field structure. However, the curves with violet hues depict the outer parts of the magnetic field of the flux rope while the blue-hued curves show the parts closer to the axis of the structure.

At earlier times the flux rope is clearly inclined towards the south as is seen in the left panel of Figure 7. As the flux rope rises, it becomes increasingly less inclined. The decrease of the inclination is clearly visible in the right panel of Figure 7. At this time, the flux rope rises upwards with a speed of $\approx1$ km s^−1^. The structure has a (minor) radius between 35 and 40 Mm at the apex, although the cross-section is only approximately circular being more elongated in the north–south direction. As the flux rope rises, also its cross section expands. Later in the simulation, between around 16 and 20 UT on June 13, the upper parts of the flux rope start to exit the simulation domain via the upper and southern boundaries.

**Figure 8:**
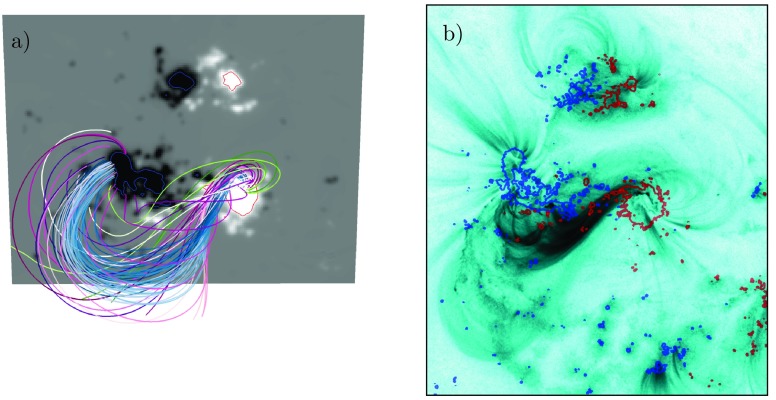
(**a**) Magnetic field lines of the flux rope at 00:00 June 13. (**b**) SDO AIA 131 Å EUV image of approximately the same region as in panel a at the time of the eruption at 13:15 on June 14. The *colorbar* in the AIA image has been inverted as compared to the standard one. The *red (blue) curves* show contours of $+(-)~500$ G.

In Figure 8, a comparison between a selection of the magnetic field lines of the flux rope (selected such as to highlight the approximate size and shape of the flux rope) at 00 UT on June 13 and the EUV coronal observation provided by the AIA/SDO 131 Å channel is shown. For the comparison, the time for the AIA observation is selected from the start of the eruption (13:15 UT on June 14) when an erupting sigmoidal signature is clearly visible in the coronal EUV observations (Palmerio *et al.*, 2017; James *et al.*, 2017). Comparing the field lines and the sigmoidal shape, there is some correspondence between the two.

## Discussion

In this section, we discuss the results of the data-driven modeling applied to NOAA AR11504.

### Photospheric Injection

In Section 4.2 we presented the results of the optimization-based procedure for determining the photospheric electric field from a sequence of processed HMI vector magnetograms. The close match of the energy injection as a function of time between the reference DAVE4VM estimate and the two assumptions for the non-inductive electric field show that the optimization is robust and able to reproduce the energy injection despite having only a single free, and constant, parameter. On the other hand, setting the non-inductive component to zero underestimates the energy injection, resulting in $\approx3.3$ times less energy injected during the considered time period.

While both assumptions for the non-inductive component are able to capture the reference energy injection computed using DAVE4VM, the injection of relative helicity shows distinct differences. For the $U$-assumption, the injection time-profile (Figure 3, yellow curve) follows the reference DAVE4VM result at all times to within $\approx75 \%$, while the $\Omega$-assumption heavily overestimates the injection. Similar conclusions, both for the energy and relative helicity injection, were also reached by Lumme, Pomoell, and Kilpua (2017) when analyzing the evolution of a different active region, NOAA AR11158. In that case, however, the $U$-assumption underestimated the relative helicity injection by $\approx34\%$. The result for the case when the non-inductive component is set to zero is also similar to that of Lumme, Pomoell, and Kilpua (2017): the relative helicity is significantly smaller compared to the other inversion methods. For AR11504, the relative helicity injection with this zero-assumption even changes the sign becoming negative after about two days from the start of the simulation. Based on our electric field inversions for AR11504 and AR11158, our conclusion is therefore that the non-inductive component cannot be neglected when attempting to accurately capture the energy and relative helicity injection of an active region. This is consistent with previous findings, *e.g.*, Fisher *et al.* (2010), Cheung and DeRosa (2012), Kazachenko, Fisher, and Welsch (2014).

Figures 2 and 3 also show the injection for the sim-dataset (dotted curves) that was further processed for use in the coronal simulation, mainly by smoothing and rebinning the magnetograms. As is visible in the plots, this additional processing step results in a consistent decrease of the injections both of energy and of relative helicity. The changes between the $\mathrm{sim}$- and $\mathrm{opt}$-datasets for the energy injection can be considered minor (total energy injection reduced at most by 22%) when taking into account estimates of errors involved in the inversion process. For instance, Kazachenko *et al.* (2015) estimate the errors in the computation of the Poynting flux for their electric field inversion of AR11158 to be $\pm14$% due to noise in the vector magnetogram data, but they rise to $\pm29$% when taking into account method-related uncertainties. On the other hand, for the relative helicity injection the changes are more substantial. For instance, for the $U$-assumption, it takes more than 24 hours longer for $10^{27}$ Wb^2^ to be injected with the $\mathrm{sim}$-dataset than for the $\mathrm{opt}$-dataset. The change in total relative helicity injected for the entire time window is about 50% larger for the $\mathrm{opt}$-dataset. For the zero-assumption, the changes due to the additional processing are more dramatic, with the relative helicity becoming smaller by more than an order of magnitude. Additionally, the change of sign occurs approximately 36 hours earlier in the processed $\mathrm{sim}$-dataset. These results suggest that not only does neglecting the non-inductive component result in severely reduced energy and relative helicity injection for this active region, but also that the zero-assumption is more sensitive to the magnetogram processing as compared to the other assumptions in terms of the relative helicity injection.

### Effective Photospheric Fluxes

The total magnetic energy content as a function of time in the coronal simulation volume, presented in Section 4.2 and Figure 4, displays an intriguing discrepancy from the photospheric energy injection shown in Figure 2. First, the time-profiles of the coronal energy do not show the same relation for the three simulations as the energy injection. Why is the energy in the $\Omega$- and $U$-simulations clearly different despite the photospheric injection being comparatively similar? Second, not only the temporal profiles are different, but there is an apparent violation of energy conservation as well. Examining the $\Omega$-simulation, the corona has gained $\approx5 \times10^{26}$ J of energy when comparing to the initial potential field. However, the energy injected by the $\mathrm{sim}$-dataset is $\approx2.5 \times10^{26}$ J, a factor of two smaller. How can the energy in the coronal volume be larger than the energy that is put into the volume?

The reason for the observed behavior is that the Poynting and relative helicity fluxes through the bottom boundary in the simulation are different from the same quantities computed from the input $\mathrm{sim}$-dataset. This is a result of the fact that the horizontal component of the magnetic field in the simulation does not evolve according to the horizontal component in the input $\mathrm{sim}$-dataset. The evolution of the horizontal components in the simulation is determined not only by the imposed photospheric horizontal electric field but also includes a contribution from the magnetofrictional electric field; there is *no* constraint that would force the horizontal magnetic field to evolve as in the $\mathrm{sim}$-dataset. This is a direct consequence of Faraday’s law, which introduces derivatives in the normal ($z$) direction in the expressions of $\partial_{t} B_{x}$ and $\partial_{t} B_{y}$, but not $\partial_{t} B_{z}$. In fact, due to the staggered co-location of the magnetic field components on the grid (Section 2.3), the simulation does not contain horizontal magnetic field components on the $z=0$ plane. Rather, the horizontal components closest to the boundary are located a half grid spacing above the photospheric boundary at $z=\Delta z/2$. In contrast, the normal component of the magnetic field in the simulation evolves according to the normal component of the $\mathrm{sim}$-dataset (though with small, random differences in $B_{z}$ that arise from the use of central difference scheme in estimating the time derivative of the magnetic field in the electric field inversion).

**Figure 9:**
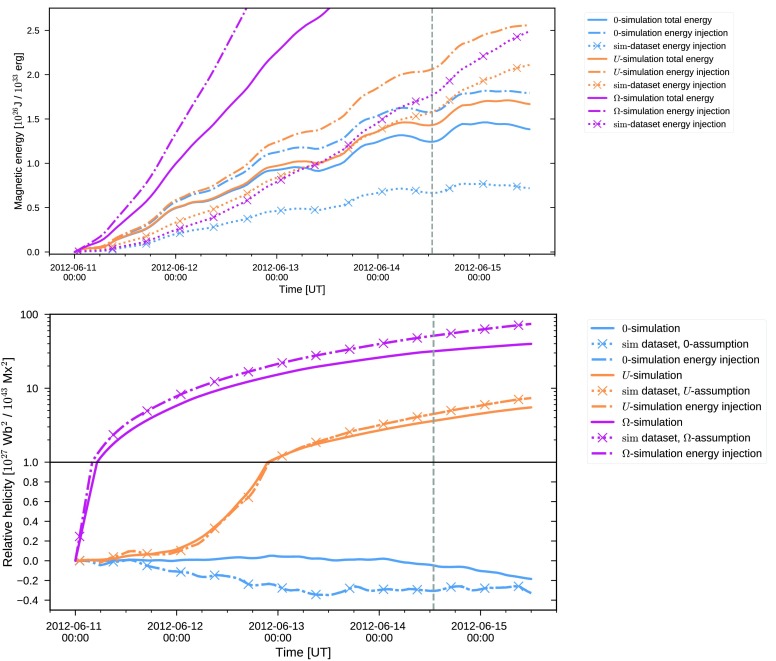
*Upper*: Total energy minus the energy of the initial potential field (*solid*), energy injection (*dashed-dotted*) in the simulation and the energy injection (*dotted with crosses*) computed from the $\mathrm{sim}$-dataset. *Lower*: Total relative helicity in the coronal volume (*solid*), injection of relative helicity in the simulation (*dashed-dotted*) and relative helicity injection computed from the input magnetograms (*dotted with crosses*).

In the upper panel of Figure 9, we compare the evolution of the total amount of energy in the simulation volume relative to the potential energy at $t=0$ (solid curves), energy injection from the photosphere in the simulation (dash-dotted curves) and the energy injection computed from the $\mathrm{sim}$-dataset (dotted curves with crosses). The horizontal magnetic field components at the photosphere in the simulation were obtained by linear extrapolation from the first two grid layers in the $z$-direction. The figure shows that there is a correspondence between the rate of energy injection and accumulation of total energy in the simulation for all assumptions. Differences are to be expected due to the possible energy flux out of the domain, as well as due to the magnetofrictional relaxation and the Ohmic diffusion (Section 2), which both lower the total magnetic energy. The energy injection as it appears in the simulation is, however, significantly different from the input dataset. The lower panel of Figure 9 plots the relative helicity in the same manner as the left panel. In this case, the injection computed from the $\mathrm{sim}$-dataset and from the photospheric magnetic field realized in the simulation agree (the dotted curve is almost indistinguishable from the dash-dotted curve as they lie almost exactly on top of each other). This can be understood by studying Equation 18: since $B_{z}$ (and thereby ${\boldsymbol{A}}_{\mathrm{p}}$) as well as the input electric field should be identical, it is expected that the realized injection of relative helicity does not change.

These results suggest an important phenomenon to take into account when performing data-driven simulations: a non-linear response of the input data in the simulation causes the effective energy fluxes to be different from the input data in a non-trivial fashion. One possible significant contributor to this is the simplification of using a potential field as the initial condition for the coronal simulation. Effectively this results in the horizontal components of the magnetic field to be inconsistent with the input dataset immediately from the start of the simulation (this could be improved using NLFFF extrapolation as the initial condition instead). However, it remains unclear why the response is so different for the two non-inductive components. It also cannot be ruled out that the magnetofrictional relaxation itself also contributes to the unexpected response of the simulation. Therefore, we conclude that computing the injection both from the input dataset and as it appears in the simulation is an important benchmark when performing data-driven simulations.

### Response of the Coronal Magnetic Field to the Driving Electric Field

The coronal magnetic field, driven by the three different electric fields, experiences a different evolution in each simulation as described in Section 4.3. In the case of the zero and $U$-simulations, the coronal evolution is similar until approximately June 13, the time depicted in Figure 6. Figure 9 shows that this is not surprising: the difference in the total magnetic energy and relative helicity injections is small up until approximately June 12, and thereafter the differences start to accumulate and become visible in the structure of the magnetic field. After this time, the relative helicity injection increases steadily in the $U$-simulation while remaining small in the 0-simulation. On the other hand, the energy evolves similarly in both simulations, and at the end of the run the $U$-simulation contains only some $14 \%$ more total magnetic energy than the zero-simulation. Despite this, the coronal magnetic field appears clearly more sheared at the end of the $U$-simulation. This can be understood in terms of the larger proportion of the energy contained as free energy in the $U$-case (≈6 times more free energy relative to the total magnetic energy). The $\Omega$-simulation behaves differently from the other two simulations. While the total energy injection (Figure 9, upper panel) is noticeably larger for this simulation, this fact appears not to solely be responsible for the difference in the configuration of the coronal magnetic field. For instance, in the $\Omega$-simulation at approximately 12:00 on June 12 the corona contains roughly the same amount of total magnetic energy as the $U$-simulation at the end of the run. However, the magnetic field in the $\Omega$-simulation appears much more sheared, with indications of the flux rope already present. This is reflected in the total relative helicity content: at this time, the $\Omega$-simulation contains roughly twice the amount of relative helicity as compared to the $U$-simulation at the end of the run.

These results suggest that relative helicity is an important metric for understanding the coronal magnetic field structure also in a data-driven context. For our simulations, the relative helicity enables one to more readily differentiate between magnetic field configuration compared to the total magnetic energy. In other words, the coronal evolution appears to be significantly different in spite of relatively similar total energy injections taking place.

Why does only the $\Omega$-simulation produce an eruption? Employing the hypothesis above, an explanation can be given in terms of the relative helicity. At the time when the flux rope appears to start to rise, the $\Omega$-simulation contains more relative helicity than the $U$ and zero-simulations do during their entire evolution. Thus, our results are compatible with the hypothesis that a threshold value for the relative helicity needs to be reached before an eruption is produced. Recently, the idea that relative helicity-based proxies rather than energy-based proxies more accurately predict the onset of eruptions has been emphasized based on idealized numerical simulations (Pariat *et al.*, 2017; Zuccarello *et al.*, 2018). Our data-driven results are consistent with this picture. However, we should point out that our results are also compatible with a similar threshold value in the ratio of the free to total magnetic energy, which also differentiates eruptive simulations from the non-eruptive ones (see Figure 4, lower panel). A more detailed discussion of such eruption proxies would necessitate more simulations with different (non-inductive) electric fields to be performed.

In the $\Omega$-simulation, a large coherent flux rope was formed. Quickly after formation, the flux rope started to rise and exited the simulation domain. This eruption process is depicted in Figure 7. A noteworthy aspect of the process of the rising of the flux rope is the speed at which it takes place: only approximately 1 km s^−1^, which is similar to typical velocities in the photosphere. This slow rise is a direct consequence of the magetofrictional method in which the velocity is given by the Lorentz force and does not include terms related to the plasma dynamics.

In Figure 4 (right panel), the ratio of free energy to the total magnetic energy is shown. As noted previously, the $\Omega$-simulation shows a markedly different behavior from the other two simulation runs, with the increase of the free energy ratio halting during the first half of June 12. This time in the simulation corresponds to the time when the first indications of a coherent flux rope structure is seen. Similarly, the saturation of the free energy ratio takes place at around June 13, 12 UT which coincides roughly with the time that the flux rope has reached the higher corona and starts to exit through the boundary. Thus, the saturation of the free energy ratio is due to a balance between the free energy exiting the domain and being injected at the photosphere.

Figure 8 presents a comparison between the coronal magnetic field at the time that the flux rope starts to rise in the simulation (panel a) and the AIA 131 Å EUV image (panel b). As can be seen, there is a correspondence between the magnetic field that constitutes the flux rope in the model and the moving S-shaped structure in the EUV image. Noteworthy is the difference in time between the two: the snapshot from the simulation is taken at 00 UT on June 13, while the observation was made on June 14 at 13:15 UT. In other words, the $\Omega$-simulation produced an eruption with characteristics similar to that of the actual observed one more than one and a half days in advance. It is, however, important to stress that linking the simulated flux rope and its lift-off to the eruption on June 14 is not self-evident. Contributing to this is the ambiguity in defining the time of eruption in the magnetofrictional simulation due to the slow eruption dynamics. Moreover, AR 11504 produced a flare with an accompanying CME on 13 UT, June 13 (see, *e.g.*, Srivastava, Mishra, and Chakrabarty, 2018). With the runs presented in this work we cannot rule out the possibility that the simulated flux rope represents the earlier eruption. The AIA 131 Å observations of the June 13 eruption show several similar features to the June 14 event. In particular, a large-scale sigmoidal structure with a southward inclination is seen to erupt, suggesting the two eruptions to be partly homologous.

In a recent study, James *et al.* (2018) performed a static non-linear force-free extrapolation of the same active region 11504 approximately one hour before the start of the eruption. Similar to our results, the authors report a large coherent flux rope structure with a distinct southward extension. The size of the flux rope is also similar to ours, reporting a minor radius of 35 Mm which is close in size to the 35 – 40 Mm radius that we find when the apex is roughly at the same height as in their extrapolation. As noted above, these results are obtained more than one day in advance of the time of the NLFFF extrapolation. James *et al.* (2018) also note that the total relative helicity in their extrapolation volume is $2.4 \times10^{27}$ Wb^2^. Referring to Figure 9, this value is obtained in the $U$-simulation at June 13 20 UT, and in the $\Omega$-simulation already on June 11, 12 UT. For the reference DAVE4VM estimate, this amount of relative helicity injection is reached at June 13 02 UT. Assuming that the value reported by James *et al.* (2018) would be the correct value, DAVE4VM therefore overestimates the injection, whereas the $U$-simulation is in close agreement. Nevertheless, the magnetic field structure at the time of the eruption is markedly different in the $U$-simulation than the one presented in James *et al.* (2018). Note, however, that the relative helicity content in NLFFF extrapolations has been found to be sensitive to choices related to magnetogram size and magnetogram processing methods in addition to the extrapolation methodology (DeRosa *et al.*, 2015).

### Role of the Non-inductive Electric Field

The results presented in Section 4 present a coherent picture: the non-inductive component provides a significant contribution to the total photospheric electric field. The results of Section 4.1 show that in order to accurately capture the energy and relative helicity injection the non-inductive electric field cannot be neglected. This significance carries over to the evolution of the coronal magnetic field: it is vastly different depending on the details of how the non-inductive component is specified, even if the energy injection is closely the same.

In a recent study, Yardley, Mackay, and Green (2018) performed data-driven magnetofrictional modeling to study the evolution of NOAA AR 11437. The study reports a good correspondence between the main coronal features of the active region and the model. The authors also performed a parameter study in which they varied among other quantities the additional injection of relative helicity. This was done by adding a non-inductive component to the driving electric field. The authors find that varying the injection of relative helicity does not change the coronal field considerably.

The results of Yardley, Mackay, and Green (2018) are not necessarily in contradiction with our findings. This is because the amount of extra relative helicity that they inject to the simulation remains rather small. In our results, the extra relative helicity required to produce significant changes is more than tenfold (*e.g.*, comparing the zero and $U$-simulations), whereas in Yardley, Mackay, and Green (2018) the increase in injection remains below a factor of ≈ 3 – 5. Another significant difference relates to the active region itself: AR 11504 in our study undergoes significant flux emergence and produces several M-class flares and multiple CMEs, while AR 11437 experiences a modest flux emergence and produces only C-class flares. It is possible that the non-inductive electric field and hence also the relative helicity injection is less pronounced for active regions that are in their decaying phase such as AR 11437 is during a significant portion of their simulation. If so, it is possible that the inductive electric field in such cases is more dominant and sufficient to produce eruptive structures. Studying active regions in different phases of their evolution can therefore provide insights into the more precise role of the non-inductive electric field in energizing magnetic field structures that lead to eruptions. It is, however, important to note that the electric field inversion employed in this study uses solely vector magnetogram data as input. Capturing the emergence process is important in order to accurately determine the non-inductive electric field. Employing additional data, for instance Doppler measurements (as in the PDFI method, Kazachenko, Fisher, and Welsch, 2014), in the inversion process provides a potential future avenue for achieving a more accurate assessment of the role of the non-inductive electric field.

## Summary and Conclusions

In this work, we performed time-dependent data-driven magnetofrictional modeling of NOAA AR 11504. First, the photospheric electric field was inverted using as input a time sequence of HMI vector magnetograms. To fully constrain the electric field, *ad hoc* assumptions for the non-inductive component were employed. This procedure was carefully conducted by optimizing the free parameters in the assumptions to reproduce the injection of total magnetic energy as provided by an independent estimate, DAVE4VM. In doing so, two distinct *ad hoc* assumptions were considered. For both cases, the resulting electric field was able to capture the total energy injection as a function of time to good agreement, although only a single temporally and spatially constant free parameters was used in the *ad hoc* assumption. The case when the non-inductive component was set to zero clearly underestimated the energy injection. In contrast to the energy, the two *ad hoc* assumptions produced vastly different injections of relative helicity. Again, neglecting the non-inductive component resulted in a severe underestimation of the relative helicity injection.

Second, the inverted photospheric electric fields were used as input to a time-dependent model of the coronal magnetic field. The resulting evolution was found to depend on the input electric fields. This was the case despite all electric fields producing the same evolution of the normal component of the magnetic field, and despite the total energy injection being similar for the optimized electric field datasets used as input to the simulations. The simulation employing a purely inductive electric field and the simulation that most closely followed the reference estimate DAVE4VM produced comparative results. However, only the simulation with the highest relative helicity and free energy content produced a flux rope that then erupted, largely in agreement with the observed eruption and with a previously reported extrapolation result of the same active region.

Our simulation results clearly show that a detailed specification of the non-inductive electric field is crucial for capturing the evolution of the coronal magnetic field. In other words, it is not enough that the electric field reproduces the observed evolution of the normal component of the magnetic field. Furthermore, it is not enough that in addition the total energy injection is correctly reproduced. In our results, we found that the relative helicity as well as free magnetic energy injection contribute significantly to the coronal response. Thus, methods to constrain the non-inductive component, either by using additional observational datasets or via theoretical arguments, are vital in order to improve the realism of data-driven time-dependent modeling.

In this work, the electric field used as the reference for optimizing the total energy injection was provided by DAVE4VM. In recent years other methods that incorporate additional datasets have been considered, in particular the PDFI method of Kazachenko, Fisher, and Welsch (2014), which employs Doppler velocity measurements. Employing a second independent estimate, *e.g.* based on PDFI, would be important for our optimization-based approach. However, for example for the case of NOAA AR11158, DAVE4VM and PDFI provide comparative fluxes of total magnetic energy and relative helicity (Lumme, Pomoell, and Kilpua, 2017). Employing different electric field inversion procedures will be subject of future efforts.

An important effect, discussed in Section 5.2, is that the horizontal components of the magnetic field at the photosphere in the simulation do not follow those used as input in the electric field inversion. As a result, the effective photospheric energy injection differs in the simulation from the one computed in the inversion process. To a degree this discrepancy is due to employing a potential field extrapolation as the initial condition for the simulation, which introduces photospheric ${\boldsymbol{B}}_{\mathrm{h}}$ components inconsistent with the observations immediately in the beginning of the simulation. However, the horizontal photospheric electric field used as input plays a crucial role as well. This suggests that using a more appropriate initial condition, as provided by *e.g.* a non-linear force-free extrapolation is necessary in particular for cases where the active region already contains significant flux at the start of the simulation.

A striking result of one of our time-dependent modeling results employing energy-optimized electric fields is the dynamics of the magnetic field when an eruptive configuration is produced. In that simulation, a flux rope structure is self-consistently generated and its dynamics is consistent with the observed eruption as well as with a non-linear force-free extrapolation computed one hour before the eruption. Moreover, the flux rope structure is produced more than one day in advance before the actual eruption occurred. This result suggests that our time-dependent data-driven model is able to provide a meaningful approximation of the dynamics of the coronal magnetic field including the formation of flux rope structures and their subsequent destabilization when the photospheric injection in the model exhibits the suitable observed trends. The degree to which this result holds for other active regions as well as what the precise conditions for obtaining realistic coronal dynamics remain to be addressed in future work. In addition, a method to determine the stability of the magnetic field structure in order to more rigorously determine the time of eruption is required. Nevertheless, our results suggest that time-dependent data-driven simulations offer a valuable tool for modeling the evolution of coronal magnetic fields with a potential for meaningful predictions of the production of eruptive structures.

## Electronic Supplementary Material

Below is the link to the electronic supplementary material.

(MP4 50.7 MB)
